# ICVAE: Interpretable Conditional Variational Autoencoder for De Novo Molecular Design

**DOI:** 10.3390/ijms26093980

**Published:** 2025-04-23

**Authors:** Xiaqiong Fan, Senlin Fang, Zhengyan Li, Hongchao Ji, Minghan Yue, Jiamin Li, Xiaozhen Ren

**Affiliations:** 1 School of Artificial Intelligence and Big Data, Henan University of Technology, Zhengzhou 450001, China; fxq@haut.edu.cn (X.F.); 231210100521@stu.haut.edu.cn (M.Y.); jiaminli@stu.haut.edu.cn (J.L.); 2 Agricultural Genomics Institute at Shenzhen, Chinese Academy of Agricultural Sciences, Shenzhen 518120, China; d22092100360@cityu.mo (S.F.); liz913925@gmail.com (Z.L.); jihongchao@caas.cn (H.J.); 3 State Key Laboratory of Crop Stress Adaptation and Improvement, School of Life Sciences, Henan University, Kaifeng 475001, China

**Keywords:** molecular generation, variational autoencoder, drug discovery

## Abstract

Recent studies have demonstrated that machine learning-based generative models can create novel molecules with desirable properties. Among them, Conditional Variational Autoencoder (CVAE) is a powerful approach to generate molecules with desired physiochemical and pharmacological properties. However, the CVAE’s latent space is still a black-box, making it difficult to understand the relationship between the latent space and molecular properties. To address this issue, we propose the Interpretable Conditional Variational Autoencoder (ICVAE), which introduces a modified loss function that correlates the latent value with molecular properties. ICVAE established a linear mapping between latent variables and molecular properties. This linearity is not only crucial for improving interpretability, by assigning clear semantic meaning to latent dimensions, but also provides a practical advantage. It enables direct manipulation of molecular attributes through simple coordinate shifts in latent space, rather than relying on opaque, black-box optimization algorithms. Our experimental results show that the ICVAE can linearly relate one or multiple molecular properties with the latent value and generate molecules with precise properties by controlling the latent values. The ICVAE’s interpretability allows us to gain insight into the molecular generation process, making it a promising approach in drug discovery and material design.

## 1. Introduction

From ancient times to the present, human beings have been suffering from diverse diseases, and they continue to find corresponding medicines that can treat diseases. Until the end of the nineteenth century, the screening of new drugs was achieved by a trial-and-error method. However, this method requires immense manpower and financial resources and suffers from low efficiency and high uncertainty. In the past few years, the integration of machine learning predictions in the development of bioactive compounds has become an essential aspect of contemporary drug discovery efforts [1,2]. The utilization of deep learning techniques for de novo drug design, such as molecular generation, has become increasingly popular due to their potential to accelerate the discovery and optimization of novel ligands with desirable therapeutic properties [3,4].

A plethora of generative models based on machine learning have been developed and customized for the specific goal of de novo design. Some promising outcomes have unequivocally showcased the potential of these approaches. For example, the deep generative model called generative tensorial reinforcement learning (GENTRL) was used for optimizing synthetic feasibility, novelty, and biological activity, which enabled the discovery and synthesis of a potent inhibitor of discoidin domain receptor 1 (DDR1) within 21 days [5]. Another model named MproI-GEN was used for designing potential inhibitors against SARS-CoV-2 Mpro, which provided a list of candidates which passed quantum chemical screening [6].

Sequence-based recurrent neural networks (RNNs) [7,8,9,10,11,12,13,14,15], variational autoencoders (VAEs) [16,17,18,19,20,21,22,23,24], reinforcement learning (RL) [25,26,27,28,29,30], and generative adversarial networks (GANs) [31,32,33,34,35] are among the many machine learning frameworks and models currently available for molecular generation. The core idea of the VAE revolves around using an encoder to understand the intrinsic molecular distribution and a decoder to generate new molecules sampled from this distribution. Within the VAE framework, introducing randomness into the molecular distribution contributes to enriching the variety of generated molecules. The encoder of the VAE converts one-hot encoded SMILES vectors or molecular graph into latent vectors. These latent vectors are subsequently exposed to controlled noise to generate original SMILES or molecular graph, facilitated by the VAE’s decoder. VAEs possess a distinct attribute in their capability to acquire a continuous and organized latent space. This latent space can be manipulated to create molecules possessing specific properties by combining it with optimization algorithms, such as Gaussian process [19] or Bayesian optimization [36].

One potential issue is that the latent space may be highly entangled, meaning that each dimension of the latent space cannot be interpreted independently of the others. This makes it challenging to understand how changes in a specific dimension of the latent space will affect the generated molecule. In the realm of interpretable machine learning (ML) for molecular design, Leguy et al. proposed the EvoMol approach [37], which utilizes an evolutionary algorithm to systematically construct molecular graphs in a sequential manner. This process of building can be effectively traced, allowing for the generation of chemically meaningful visualizations. Such an interpretable methodology holds promise for refining the exploration process in molecular design. Krishnan et al. [38] introduced a machine learning strategy aimed at autonomously designing protein kinase inhibitors. Their method combines a Variational Autoencoder (VAE) with a novel cluster-based perturbation technique, enabling a systematic exploration of the chemical latent space. Notably, this approach promotes the grouping of molecules with comparable structures in the latent space. Moreover, interpolating between molecules in this space enables the seamless transformation of molecular structures and associated properties. Noutahi et al. introduced LaPool (Laplacian Pooling) [39], a hierarchical graph pooling technique that is data-driven and interpretable. This inventive method efficiently merges node features and graph structures to improve molecular representation. By utilizing the graph Laplacian, LaPool identifies crucial graph substructures and promotes the grouping of nodes from the same substructure within the condensed graph.

Together, the previously mentioned approaches are aligned in their pursuit of enhancing the interpretability of molecular graph generation. These methodologies are focused on utilizing machine learning techniques to craft molecules exhibiting similar structures or substructures, while also providing a transparent avenue for refining the generative process. However, there is still a gap in property-driven latent space interpretability. Importantly, the primary objective is not to render the generative process geared towards similar structures or substructures more interpretable. Rather, its emphasis lies in enhancing the interpretability of the generative process with respect to similar properties.

**Figure 1:**
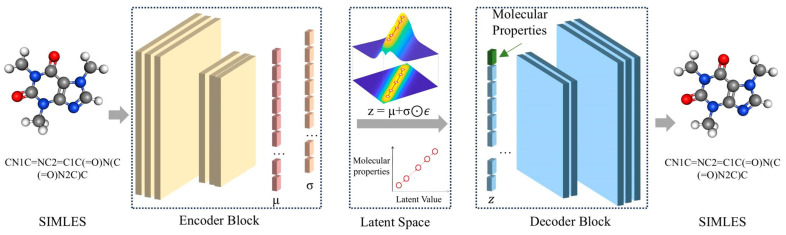
Overall architecture of ICVAE. The ICVAE consists of an encoder block, a latent space, and a decoder block. Initially, the one-hot SMILES vector and molecular properties are fed into the encoder block to derive the latent space representation of the input data. Subsequently, the decoder block generates the one-hot SMILES vector from the latent space. The key difference between the ICVAE and traditional CVAE is that the latent values of ICVAE are linearly dependent with the conditional inputs (molecular properties).

In this work, we present a novel approach to achieve interpretability in the latent space of CVAE. Our method, ICVAE, establishes a linear relationship between the conditional input and latent value (Figure 1). We evaluate various conditional inputs, including individual and pairwise property combinations. ICVAE enable the interpretation of generated molecule properties in the latent space. It makes it possible for the researchers to understand the meaning behind the latent space, e.g., the kind or the value of molecular properties. This capability supports intuitive, interactive molecular design, particularly in multi-objective settings where multiple properties must be balanced. We believe this property-centric latent space is a significant step toward bridging the gap between deep generative models and real-world applications in drug discovery.

## 2. Results

The following section outlines three experiments that were conducted to demonstrate the interpretability of ICVAE. Experiment 1 involved a comparison of the latent space of ICVAE with VAE and CVAE, demonstrating the efficacy of ICVAE in rendering the latent space explicable. Experiment 2 further showcased the versatility of ICVAE, with the model capable of mapping multiple molecular properties to the latent space. Finally, experiment 3 highlighted the ability of researchers to randomly select a point in the ICVAE latent space to generate molecules with specific target properties.

### 2.1. Workflow Summary

The ICVAE workflow consists of three interconnected phases: (1) Data Preprocessing: SMILES strings are standardized, padded with start/end tokens (maximum length = 120), tokenized into atomic symbols, and converted into one-hot vectors. Simultaneously, molecular properties are normalized into conditional labels aligned with the latent dimensions. (2) Model Training: The encoder—built with convolutional and dense layers—maps SMILES–condition pairs into a latent space where each dimension is explicitly associated with a target property. A modified loss function enforces a linear correlation between latent values (z) and property labels (c) by penalizing deviations from the relation z = τc + ϵ, where τ scales the latent range and ϵ represents stochasticity. Unlike standard CVAEs, conditional inputs are incorporated at both the encoding and decoding stages to reinforce interpretability. (3) Controlled Generation: Users specify target property values, which are directly mapped to predefined latent coordinates. The decoder then generates molecules whose latent vectors correspond to these coordinates, ensuring the desired property fidelity.

### 2.2. The Interpretability of ICVAE

**Figure 2:**
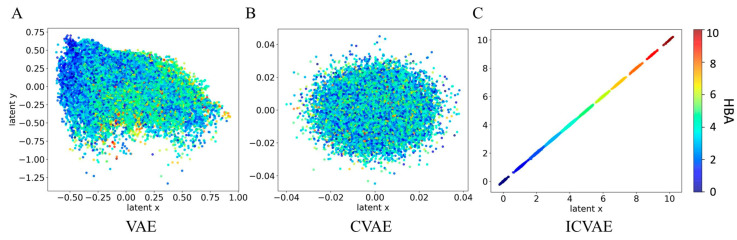
Comparison among the two-dimensional latent space of different methods in terms of their interpretability with respect to molecular HBA (hydrogen bond acceptor) property. (**A**) Two-dimensional latent spaces of VAE. (**B**) Two-dimensional latent spaces of CVAE. (**C**) Two-dimensional latent spaces of ICVAE. The horizontal and vertical axes correspond to the two dimensions of the latent vector: x represents the first dimension, while y represents the second dimension. The colormap depicts the relationship between the latent value and HBA value, where red represents higher HBA values and blue represents lower values. The VAE latent space shows some interpretability, but its distribution is non-standard and varies during training. The CVAE has a standard normal distribution, but the latent space lacks interpretability. In contrast, the ICVAE latent space exhibits a good linear relationship with the HBA value, making it more interpretable.

Figure 2 presents a comparison of the latent space constructed by ICVAE, VAE, and CVAE trained on the same dataset. Two-dimensional axes were extracted from the latent vectors to show their relationship with the HBA values. The soft label of HBA served as the conditional input for both CVAE and ICVAE. For the VAE, a multivariate right-skewed normal distribution of latent points is obtained, while the distribution of CVAE more closely resembled a multivariate normal distribution. That is because the CVAE can correspond the conditional input (HBA values) with the distributions of latent points. Therefore, ten HBA scaled values correspond to ten norm distributions and the distributions are independent of each other. This approach allows for the generation of molecules with a target property, with CVAE separating the latent space according to molecular properties to enable controllable property generation. However, the interpretability of the latent space is sacrificed, with the meaning behind each latent point remaining unknown. In other words, the relationship between the molecular properties and the latent point is unknown. In contrast, ICVAE establishes a linear relationship between the property and latent point, resulting in ten distributions in the latent space that are linearly related to ten scaled HBA values. As a result, the latent point of each HBA scale value has its own distribution, allowing for the linear interpretation of molecular properties.

### 2.3. The Latent Space of ICVAE with Single Property

**Figure 3:**
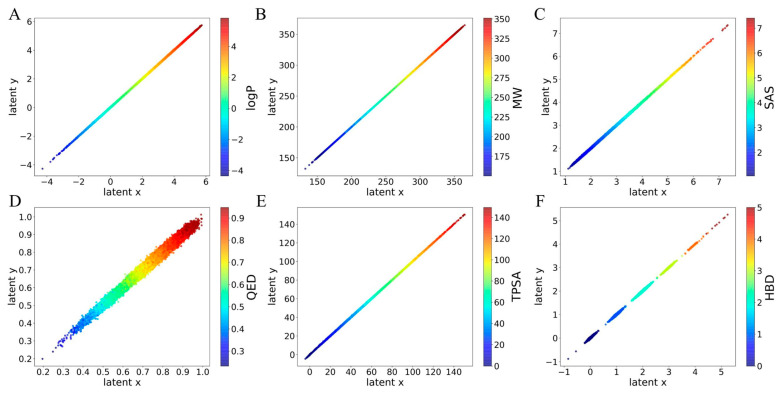
The relationship between latent space of ICVAE with single molecular property. (**A**) The relationship between latent space of ICVAE with logP. (**B**) The relationship between latent space of ICVAE with MW. (**C**) The relationship between latent space of ICVAE with SAS. (**D**) The relationship between latent space of ICVAE with QED. (**E**) The relationship between latent space of ICVAE with TPSA. (**F**) The relationship between latent space of ICVAE with HBD. The horizontal and vertical axes correspond to the two dimensions of the latent vector: x represents the first dimension, while y represents the second dimension. The value of the color bar represents each molecular property value.

We present a comprehensive analysis of the interpretability of the ICVAE, which enables a linear correlation between the latent space and molecular properties. Figure 3 illustrates this relationship between the latent space and six additional molecular properties, namely MW, logP, SAS, QED, HBA, HBD, and TPSA, with each property corresponding to two-dimensional axes in the latent vector. The latent space of MW shows the strong, continuous linear relationship between the MW value and the latent space, indicating that the learned latent points can represent the MW latent value. By unraveling the neural network’s “black box”, the researchers can understand the representation of latent points learned by the network for specific molecular properties. The latent space of the other properties also shows a similar phenomenon.

Below the MW range of 150 of latent space, the latent points are discontinuous because there are very few molecules in the training dataset with a MW below 150. The latent points cannot maintain good continuity near the boundaries (minima and maxima) of molecular properties, which is attributable to epistemic rather than aleatoric uncertainty in our ICVAE. In other words, the reason for discontinuous latent space is the insufficient number of training samples with molecular features close to the boundary, and not the inherent recognition error of ICVAE. Additionally, the latent space of HBA and HBD is discontinuous and contains discrete line segments because the HBA and HBD values are discrete. In the latent space of HBD, the ICVAE erroneously converges to latent values less than zero at very few points. This is incorrect because the HBD is always greater than or equal to zero.

### 2.4. The Latent Space of ICVAE with Multiple Properties

**Figure 4:**
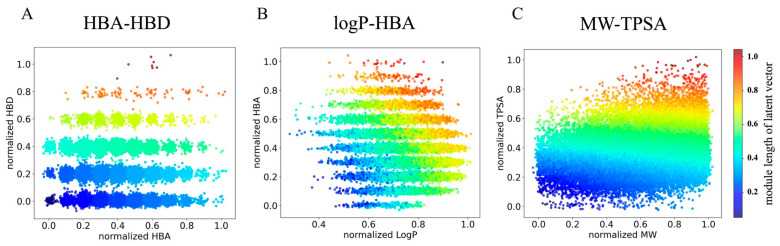
The relationship between latent space of ICVAE with multiple properties. The horizontal and vertical axes represent the two dimensions of latent x and y which are the first and second dimensions of the latent vector, respectively. (**A**) HBA-HBD. (**B**) logP-HBA. (**C**) MW-TPSA. To make two dimensions have same range, we calculate the normalized value of each property. The value of color bar represents the module length of latent vector, which is calculated by $\sqrt{x^{2}+y^{2}}$.

Furthermore, the ICVAE not only allows for the correlation of a single molecular property with the latent value, but also enables the correlation of two or more properties with the latent value. Each property corresponds to one dimension of the latent space. For example, Figure 4 shows the results of correspond latent space with two molecular properties, one at a time. The first plot is the result corresponding the HBA and HBD with the latent space. In order to give the different properties the same scale of latent space, we normalized the latent value of HBA and HBD to the range from 0.0 to 1.0. As HBA and HBD only have integer values, the two dimensions of latent space exhibit several discrete latent distributions. In the second plot, we correlated logP and HBA with the latent space, where the dimension of logP is continuous, while the dimension of HBA is discrete, which is reasonable because logP is a continuous property value. The third plot shows that the dimensions of MW and TPSA exhibit continuous value distributions in both latent dimensions, as both properties have continuous values.

### 2.5. The Performances of Molecular Generation

The performances of the ICVAE were evaluated and compared with two baseline models, VAE and CVAE, using MOSES evaluation metrics [40] to assess the quality of the generative models. The MOSES metrics include Fraction of valid (Valid), Similarity to a nearest neighbor (SNN), Fragment similarity (Frag), Novelty and Internal diversity (IntDiv), and Fraction of unique (Unique). Valid is defined using RDKit’s molecular structure parser that checks atoms’ valency and consistency of bonds in aromatic rings. The assessment involved sampling 25,000 molecules from each model under randomly chosen molecular property values. SNN is an average Tanimoto similarity between fingerprints of a molecule from the generated set and its nearest neighbor molecule in the reference dataset. Novelty is the fraction of generated molecules that pass filters applied during dataset construction. IntDiv assesses the chemical diversity within the generated set of molecules. Unique@1k quantifies ratios of unique molecules that are present within the top-k generated molecules. The results, as presented in Table 1, showed that the CVAE model achieved a notable improvement in validity, novelty, and IntDiv at the expense of reduced precision of SNN and Frag, compared to VAE. This is believed to be due to the condition input of CVAE, which limits its optimization for molecules under given conditions or molecular properties. Consequently, the CVAE model could focus more on finding molecules with enhanced validity, novelty and unique@1k, albeit possibly deviating from the distribution distance (SNN, Frag) of the test dataset.

In contrast, the ICVAE model was further optimized for finding molecules with specific molecular properties by correlating the conditions with the latent variable. As such, the ICVAE model could identify more valid and novel molecules compared to the CVAE model, as it made the conditions stronger. In essence, the ICVAE model could efficiently locate more molecules that match the given molecular properties while still achieving high validity and novelty.

### 2.6. Molecular Generation with Target Properties

The primary advantage of the ICVAE model is its ability to generate SMILES strings of molecules with desirable properties by controlling the latent value, which is equivalent to the molecular property value. To evaluate the model’s performance, we trained it on the ZINC dataset and tested its ability to generate molecules with single molecular property by changing the latent value of the single property. We varied the latent value of molecular weight (MW) from 210 to 490 at 20 intervals, logP from 0.2 to 5.8 at 0.4 intervals, QED from 0.05 to 0.61 at 0.04 intervals, SAS from 1.2 to 6.8 at 0.4 intervals, TPSA from 18 to 74 at 4 intervals, HBA from 0 to 10 at 1 interval, and HBD from 0 to 5 at 1 interval. This experiment allowed us to test the model’s ability to accurately control and generate molecules with a range of desirable properties.

**Figure 5:**
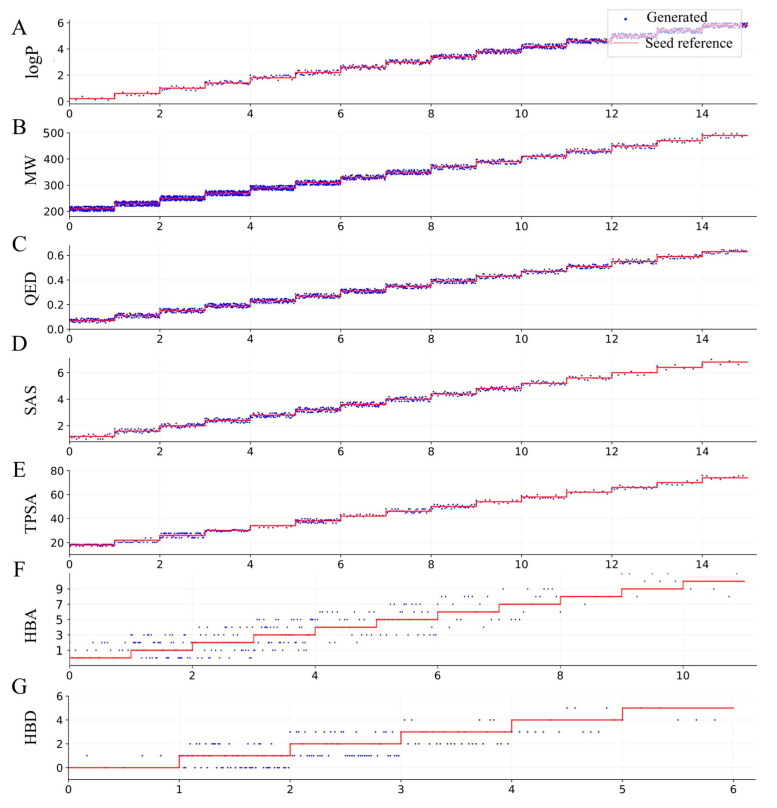
Molecular properties of valid SMILES generated by the ICVAE model with single-property condition input. The plot shows the seven calculated molecular properties, including (**A**) logP, (**B**) MW, (**C**) QED, (**D**) SAS, (**E**) TPSA, (**F**) HBA, and (**G**) HBD, of the generated molecules (blue) with given conditional latent values (red). The sampling size is equal for each conditional step.

After generating SMILES from the ICVAE model at equal sampling size for each conditional latent value, the validity of the generated SMILES was calculated using RDKit. Only the absolute error of generated properties and the target properties less than a boundary value were selected for each condition. As shown in Figure 5, a large number of valid SMILES (red) were generated by the ICVAE model for single-property condition input (blue), demonstrating the model’s ability to precisely control the single molecular property by changing the latent value of the single property.

This experimental process brings confidence to the practical application of the ICVAE method. For example, using ICVAE, medicinal chemists can generate molecules by regulating the molecular property values, analyze the generated molecular structures and evaluate their potential value in practical applications, thereby interactively generating and optimizing the molecular property values.

Upon examining Figure 5, we observed that generating molecular properties beyond a certain boundary value was challenging in some cases. Specifically, we found it difficult to generate molecules with MW values exceeding 400 and SAS values greater than 12. This may be due to a lack of training samples in these value intervals. Additionally, we observed that generating molecules with desirable HBA and HBD values was more difficult than generating other properties. This is a known issue with other conditional neural network models for molecular generation, such as cRNN [23].

**Figure 6:**
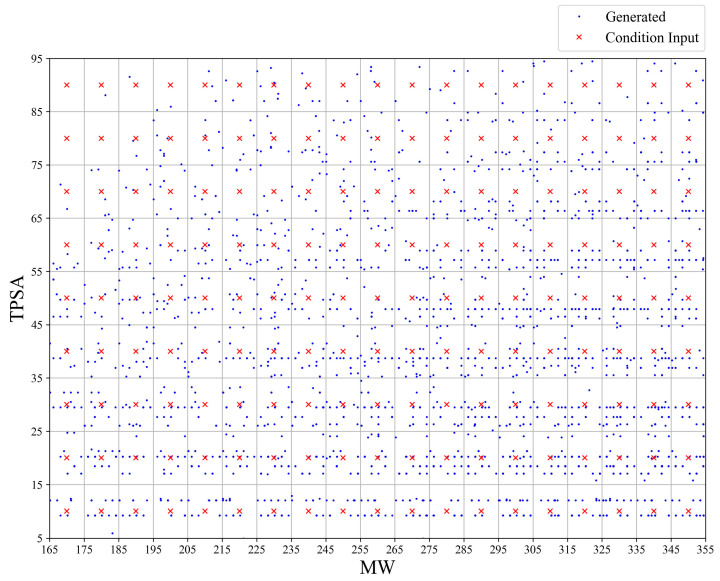
Molecular properties of valid SMILES generated by the ICVAE model with two-property condition as input. The plot shows the MW and TPSA value of the generated molecules (blue) with given conditional input (red). The x axis represents the MW value, and the y axis represents the TPSA value. The sampling size is equal for each conditional step.

To further validate the generation ability of the ICVAE model, the model with two different properties (MW and TPSA) as input condition is also trained and tested by changing the latent value of two properties. Specifically, for the two-properties-generation experiment, the latent value of MW is varied from 170 to 350 at 10 intervals, and the latent value of TPSA is varied from 10 to 90 at 10 intervals. Please note the MW and TPSA are varied simultaneously at each condition step. Figure 6 shows the molecular properties of valid SMILES generated by the ICVAE model input with the two-property condition (MW and TPSA), demonstrating the model’s ability to precisely control the generation of molecules with two molecular properties simultaneously. Upon examining Figure 6, we observed that it is difficult to generate molecules with TPSA values greater than 70 and MW values below 190. Except this range, other conditions generate enough molecules with target properties.

## 3. Discussion

This study investigates the effectiveness of an interpretable molecule generation method based on Conditional Variational Autoencoders. The proposed method correlates the molecular properties with the latent values of molecular generation, making the molecular properties interpretable in the latent space. Our findings demonstrate that this method not only enables the interpretability of a single molecular property but also multiple molecular properties simultaneously. Moreover, the proposed method is shown to be highly effective and precise in generating molecular SMILES with corresponding molecular properties.

The ICVAE offers a notable benefit by enabling the encoding of high-dimensional molecular features (such as molecular properties) into latent vectors. This paves the way for efficient multi-objective optimization by constraining the values of these latent vectors. Building upon the foundation of CVAE, ICVAE enhances the interpretability of the latent vector by establishing a correlation between molecular properties and the latent vector itself. This advancement allows for the visualization and elucidation of the multi-objective optimization process.

### Limitations and Future Work

While the ICVAE successfully enhances interpretability within the latent space of CVAE, it encounters challenges in generating a substantial portion of molecules when the training dataset is limited in size. Looking ahead, our focus will involve investigating the fusion of few-shot learning methodologies with ICVAE. This integration aims to amplify the generation capability of ICVAE, particularly in scenarios where the availability of training samples is restricted. This exploration holds the potential to bolster the model’s capacity to generate diverse and meaningful molecular structures even with sparse training data.

## 4. Materials and Methods

### 4.1. Dataset

The dataset used in this paper is the publicly available ZINC dataset [41]. Instead of using the total dataset to feed into our ICVAE, we randomly selected 500,000 molecules. This is because increasing the used molecules did not increase the performance of our model. A total of 70% of the selected molecules were used for training, and the rest were used for testing. The five molecular properties MW, logP, HBD, HBA and TPSA were calculated by RDKit (Version: 2024.9.4).

### 4.2. Data Processing

Our ICVAE is an extension of the CVAE model, which takes in two input data. The first input data are a one-hot encoding vector of the molecular Simplified Molecular Input Line Entry System (SMILES). This representation provides a simple way to describe the chemical structure of molecules that can be easily processed by computers. To indicate the end of the SMILES string, we added an ‘E’ at each end of the string. Subsequently, the SMILES representations were transformed into one-hot encoding vectors, each with a standard size of 33 × 120. Here, 33 denotes the encoding notation of the SMILES, and 120 represents the maximum length of the SMILES strings.

The second input data to our ICVAE model is molecular properties, which are conditional labels that the model uses to control the properties of the generated molecules. The raw labels are normalized into the conditional labels ranging from 0 to 500 for each molecular property. The wide range can make sure the ICVAE learns the distance between every single property value. After that, the conditional labels are not only fed with the input SMILES one-hot encoding vectors, but also with the latent vectors to reconstruct the SMILES one-hot encoding vectors. The way of feeding the conditional vector both to the input and latent vector is the same as the CVAE. By appending the conditional vector at both the encoding and decoding stages, the CVAE is ensuring that the generated data adhere to the desired condition from the very beginning (during encoding) and throughout the generation process (during decoding). This helps in producing data samples that are not only realistic but also satisfy specific requirements.

Note that including the conditional vector in both the input and latent vector is crucial for producing molecules with desired properties for CVAE. Building upon the foundation of CVAE, ICVAE establishes a link between the latent vector’s value and the conditional input. This connection enhances the interpretability and control of the generative process by modifying the latent value. Although the latent space of ICVAE can retain its linearity even without the added conditional vector, the capability to generate molecules with target properties would be forfeited.

### 4.3. Algorithm of ICVAE

**Figure 7:**
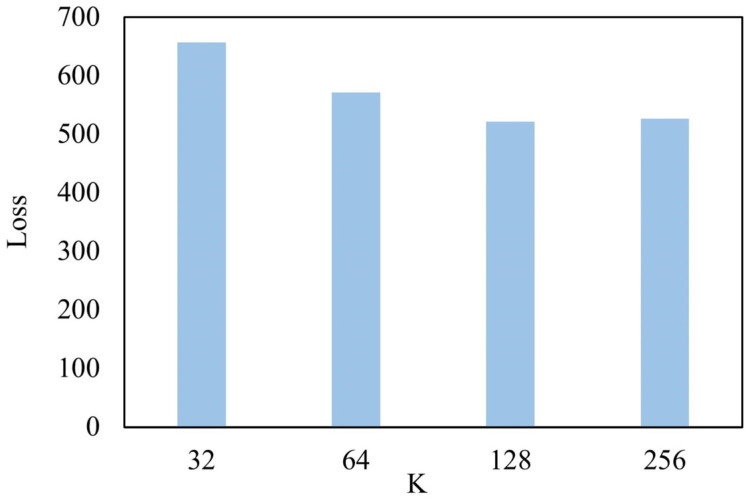
The loss value of test dataset under different K values.

The architecture of our ICVAE model is illustrated in Figure 1, which closely resembles the CVAE. It consists of an encoder block, a latent space, and a decoder block. The encoder block comprises three convolutional layers and three dense layers. Initially, the one-hot SMILES vector and molecular properties (conditional labels) are fed into the three convolutional layers which contains 16, 32, and 64 filters, respectively, with the kernel size of 11 × 3 and stride of 2. The first dense layers are added after the three convolutional layers to convert the high-dimensional outputs from the convolutional layers into the 292-dimensional hidden vectors. Then, the hidden vectors pass through the second and the third layers to obtain two K-dimensional latent vectors. The two K-dimensional latent vectors are the mean vector μ and standard deviation vector σ, respectively. The reparameterization trick is used to create the K-dimensional latent vector z:

(1) z=μ+σ⊙ϵ

where ⊙ is the Hadamard product, and ϵ is the random vector sampling from a normal distribution. In this study, we set K as 128 because the generation performance of ICVAE is greatly improved when using K from 32 or 64 to 128 and not significantly improved when using K from 128 to 256 (Figure 7). Subsequently, the decoder block symmetrical to the encoder block generates the one-hot SMILES vector from the latent vector z, taking into account the given molecular properties.

The primary distinguishing feature of the ICVAE model is its interpretable latent space. Specifically, the latent value is linearly dependent on the molecular properties. To highlight the rationale behind the ICVAE, we compared its objective functions with those of the VAE and CVAE models. The objective of the VAE is to maximize the following function:

(2) $$E_{q\left(z|x\right)\left[\log p\left(x|z\right)\right]}-D_{KL\left(q\left(z|x\right)|p\left(z\right)\right)}$$

However, the properties of molecular generation cannot be readily controlled since the latent distributions vary with different training processes in the VAE. In contrast, the CVAE incorporates an additional condition input that can guide the network to generate molecules with desirable properties. The objective of the CVAE is to maximize the following function:

(3) $$E_{q\left(z|x,c\right)\left[\log p\left(x|z,c\right)\right]}-D_{KL\left(q\left(z|x,c\right)|p\left(z|c\right)\right)}$$

where *p*(*x|c*) is the probability of generating *x* given the conditional input *c*. The CVAE provides researchers with the ability to control the molecular properties by providing input conditions ***c***. However, the latent space in the CVAE is inexplicable and implicit, making it difficult for researchers to associate specific points in the latent space with particular molecular properties. To overcome this limitation, the proposed ICVAE correlates the input conditions *c* with the latent *z*. In other words, ICVAE optimizes the latent values associated with individual molecular property by aligning them with distinct independent Gaussian distribution, each with its own specific mean. The objective of ICVAE is to maximize the following function:

(4) $$E_{q\left(z-\tau c|x,c\right)\left[\log p\left(x|z-\tau c,c\right)\right]}-D_{KL\left(q\left(z-\tau c|x,c\right)|p\left(z-\tau c|c\right)\right)}$$

where τ is the factor controlling the ratio of condition *c* to latent *z*. In the experiment, we found the best τ is to expand the range of latent conditions to [0, 500]. For example, the τ of training the ICVAE with the HBA condition input is 50.

## Figures and Tables

**Table 1:** Comparison of ICVAE and baseline models in terms of MOSES evaluation metrics of the molecules ^#^.

Model	Unique@1k↑ ^a^	Valid↑	SNN↑	Frag↑	Novelty↑	IntDiv↑
VAE	**1.000**	0.977	**0.626**	**0.999**	0.695	0.856
CVAE	0.975	0.971	0.315	0.925	0.985	0.832
ICVAE	**1.000**	**0.979**	0.339	0.843	**1.000**	**0.869**

^#^ The ZINC test set was used as a reference set for the (MOSES framework). ^a^ ↑ Represents the higher the better. Bold text indicates the best results.

## Data Availability

The dataset used to train all models are randomly selected from the ZINC dataset, which are available in https://zenodo.org/record/8278179 (accessed on 1 March 2025). The Python (Version: 3.6.13) code and the trained neural networks used in this work are available under MIT licenses at https://github.com/Xiaqiong-Fan/ICVAE (accessed on 1 March 2025).
